# Micro‐diffusely abnormal white matter: An early multiple sclerosis lesion phase with intensified myelin blistering

**DOI:** 10.1002/acn3.52015

**Published:** 2024-02-29

**Authors:** Antonio Luchicchi, Gema Muñoz‐Gonzalez, Saar T. Halperin, Eva Strijbis, Laura H. M. van Dijk, Chrisa Foutiadou, Florence Uriac, Piet M. Bouman, Maxime A. N. Schouten, Jason Plemel, Bert A. 't Hart, Jeroen J. G. Geurts, Geert J. Schenk

**Affiliations:** ^1^ Department of Anatomy and Neurosciences Amsterdam University Medical Centers, location VU Medical Center, Amsterdam Neuroscience Amsterdam the Netherlands; ^2^ MS Centrum Amsterdam, Amsterdam University Medical Centers, location VU Medical Center Amsterdam the Netherlands; ^3^ Department of Neurology Amsterdam University Medical Centers, location VU Medical Center Amsterdam the Netherlands; ^4^ Department of Neuroscience University of Alberta Edmonton Alberta Canada

## Abstract

**Objective:**

Multiple sclerosis (MS) is a chronic central nervous system disease whose white matter lesion origin remains debated. Recently, we reported subtle changes in the MS normal appearing white matter (NAWM), presenting with an increase in myelin blisters and myelin protein citrullination, which may recapitulate some of the prodromal degenerative processes involved in MS pathogenesis. Here, to clarify the relevance of these changes for subsequent MS myelin degeneration we explored their prevalence in WM regions characterized by subtly reduced myelination (dubbed as micro‐diffusely abnormal white matter, mDAWM).

**Methods:**

We used an in‐depth (immuno)histochemistry approach in 27 MS donors with histological presence of mDAWM and 5 controls. An antibody panel against degenerative markers was combined and the presence of myelin/axonal aberrations was analyzed and compared with the NAWM from the same cases/slices/regions.

**Results:**

mDAWM‐defined areas exhibit ill‐defined borders, no signs of Wallerian degeneration, and they associate with visible veins. Remarkably, such areas present with augmented myelin blister frequency, enhanced prevalence of polar myelin phospholipids, citrullination, and degradation of myelin basic protein (MBP) when compared with the NAWM. Furthermore, enhanced reactivity of microglia/macrophages against citrullinated MBP was also observed in this tissue.

**Interpretation:**

We report a new histologically defined early phase in MS lesion formation, namely mDAWM, which lacks signs of Wallerian pathology. These results support the prelesional nature of the mDAWM. We conceptualize that evolution to pathologically evident lesions comprises the previously documented imbalance of axo‐myelinic units (myelin blistering) leading to their degeneration and immune system activation by released myelin components.

## Introduction

Multiple sclerosis (MS) is a chronic degenerative and (neuro)inflammatory disease of the central nervous system (CNS) whose etiology remains debated.^1^, ^2^, ^3^ A prominent pathological feature of this disorder is the loss of axon‐enwrapping myelin sheaths both in white (WM) and gray matter, a condition that leads to the formation of the sclerotic plaques.^4^ These aberrations represent a well‐characterized final stage of myelin degeneration. However, little is known about the events occurring in seemingly normal WM (e.g., normal appearing WM, NAWM) that culminate in myelin disintegration/lesion formation. Recent investigations showed that, in comparison with controls, the MS NAWM presents with enhanced prevalence of typical myelin blisters where the myelin detaches from its enwrapped axon.^2^, ^5^ This hampers the functioning of the axo‐myelinic synapse.^6^ This synapse is shown to be key to the support of axon‐myelinic functionality enabling a glutamate‐mediated coupling between myelin and its underlying axon via myelin Ca^2+^ increase.^6^ This change at the level of myelin blistering in MS is further accompanied by a number of morphologically and biochemically relevant alterations, occurring in the absence of overt inflammation, that involve: (i) nodes of Ranvier/nodal protein aberrations,^5^ (ii) altered myelin lipid polarity and carbon‐hydrogen shifts,^5^, ^7^ and (iii) myelin protein citrullination.^5^ Nodal alterations might influence axonal conduction, increasing the axonal Ca^2+^ entrance,^8^ leading to myelin lipid biochemical changes which over‐activate the protein arginine deiminases (PADs) enzymes,^9^ with consequent myelin basic protein (MBP) citrullination.^10^ The latter event, previously reported in pre‐immune demyelination stages in cuprizone mouse models of MS,^11^ can contribute to enhanced myelin debris immunogenicity^12^ and myelin lamella decompaction, a phenomenon associated with higher myelin vulnerability to protease attack^13^ and myelin‐associated glycoprotein hydrolysis‐related myelin detachment from axonal processes.^14^

Nevertheless, whether this sequence of events is also relevant to early demyelination in MS brains remains uncertain. To address this, we concentrated our analysis on patches of microscopic diffuse abnormality in the MS NAWM, which may represent subtle manifestations of ongoing demyelination. These areas, which we coined microscopically manifest diffusely abnormal white matter (mDAWM), share similarities with the classically imaging/histologically defined DAWM,^15^, ^16^ although expanding to smaller surfaces in the NAWM (tenths to tens of mm^2^). Moreover, they show an association with inner veins,^17^ blood vessels around which the myelin abnormality spans as in the most established WM lesions,^18^ and they cannot be further explained by overt Wallerian degenerative pathology^15^ or ongoing remyelination (in comparison with the frank remyelinating/remyelinated MS shadow lesions^19^). Interestingly, the mDAWM presents with pathological mechanisms previously reported in the NAWM,^5^, ^7^ including a myelin blister increase, higher presence of the myelin polar phospholipids and enhanced MBP citrullination. Altogether, the mDAWM might represent a candidate to further explore the early degenerative events connected to MS WM lesion formation, emphasizing myelin blisters as important early drivers of demyelination.

## Materials and Methods

### Donor material

**Figure 1:**
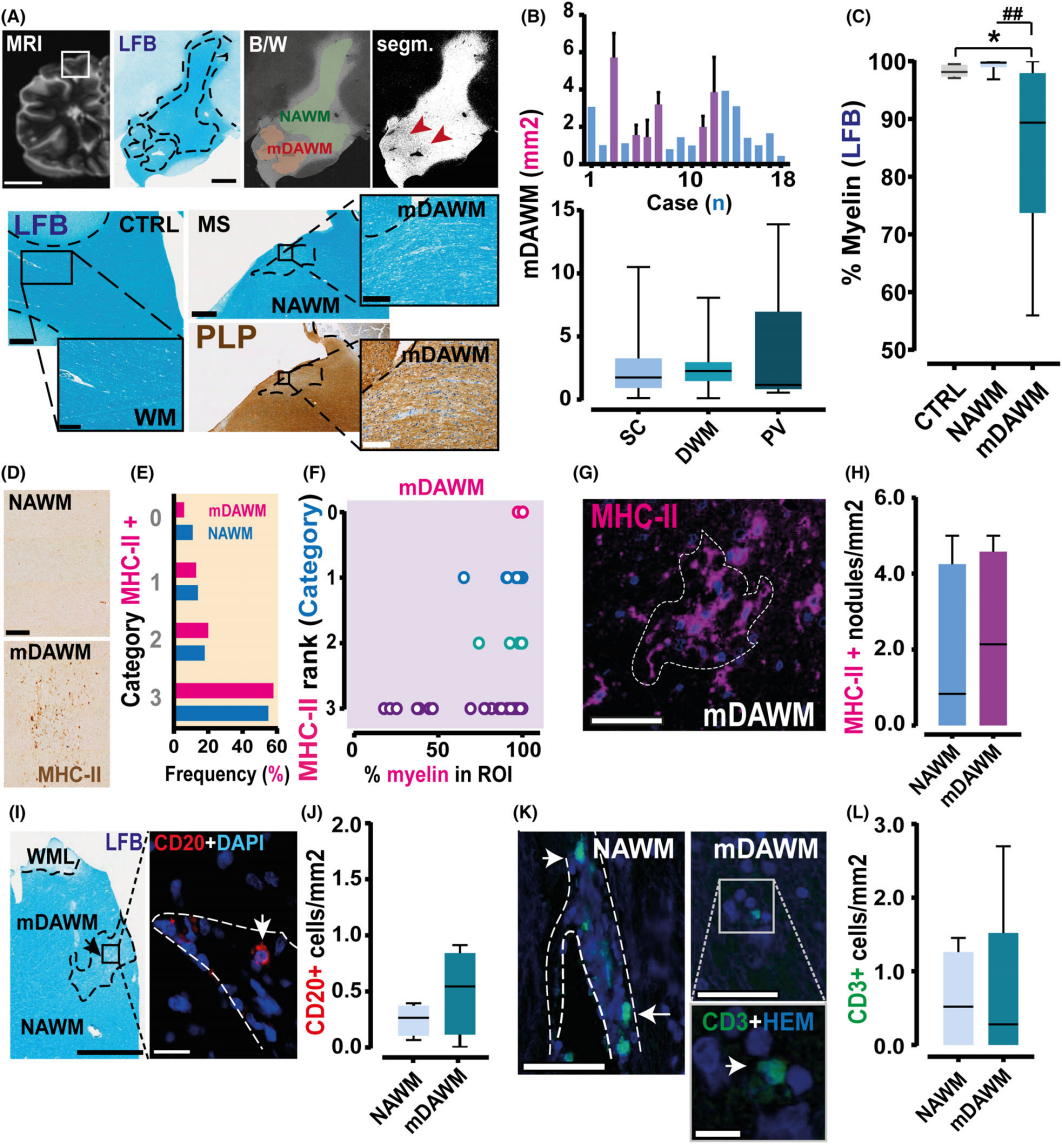
mDAWM characterization in MS tissue. (A) Left panel on top shows a 3T postmortem scan acquisition. White square: regions used for histology. Top left: microscopic image shows LFB stained tissue. The other two images refer automated mDAWM analysis (orange labeled surface). Bottom panel: LFB acquisitions from a control (CTRL, left) and an MS donor (right). PLP staining refers to the same region used for LFB staining. (B) Top graph: extension in mm^2^ of the analyzed MS mDAWM (*n* = 18). Blue bars: cases from which one block was analyzed. Purple bars: cases where multiple blocks were screened. Bottom plot: analysis stratifying the mDAWM extension according to the location in the tissue (SC *n* = 35; dWM = 29; PV = 6 regions; 2.522 ± 0.404 vs. 2.571 ± 0.319 vs. 3.726 ± 3.123 μm^2^; Kruskal–Wallis test; K = 1.128; *p* = 0.569 SC vs. dWM vs. PV). (C) Percentage of myelin inside mDAWM ROIs versus control WM and MS NAWM (*n* = 18; 99.430 ± 0.185 vs. 86.020 ± 13.060%; Wilcoxon test; W = 153.0; *p* < 0.0001; NAWM vs. mDAWM) and versus controls (*n* = 5; mean controls: 98.32 ± 0.47%; Mann–Whitney test; *U* = 18; *p* = 0.040; control WM vs. MS mDAWM). (D) Examples of microscopic acquisition of NAWM (top) and mDAWM (bottom) areas stained with antibodies against LN3 clone of MHC‐II. (E) Classification of MHC‐II reactivity on the tissue did not differ between NAWM and mDAWM (ROI n NAWM = 54; ROI n mDAWM = 43; Chi^2^ test; Chi_(3)_ = 0.553; *p* = 0.907; NAWM vs. mDAWM). (F) Spearman's correlation analysis between level of myelination in the mDAWM and microglia ranking score shows an inverse correlation (*n* = 49; Spearman r test; *r* = −0.367; *p* = 0.009; mDAWM myelin vs. MHC‐II rank). (G) Representative image of clusters of MHC‐II+ cells (inside dashed line) retrieved in MS tissue. (H) Density of MHC‐II+ clusters did not differ between NAWM and mDAWM in a sample of MS cases (*n* = 6; 1.780 ± 0.910 vs. 2.290 ± 0.920 nodules/mm^2^; Paired *t*‐test; *t*_(5)_ = 0.351; *p* = 0.740; NAWM vs. mDAWM). (I) Left images: LFB example of a mDAWM region from an MS case. Right images: same area stained using antibodies anti‐CD20. (J) Density of CD20^+^ lymphocytes between NAWM and mDAWM (*n* = 4; 0.248 ± 0.070 vs. 0.501 ± 0.191% CD20^+^ cells; Wilcoxon test; W = 6.0; *p* = 0.375; NAWM vs. mDAWM). (K) Example of CD3+ lymphocytes retrieved in NAWM and mDAWM (arrows). (L) No difference between density of CD3+ cells in NAWM and mDAWM was found (*n* = 5; 0.608 ± 0.289 vs. 0.664 ± 0.513 cells/mm^2^; Wilcoxon test; W = 2.00; *p* = 0.875; NAWM vs. mDAWM). Scale bars (from left to right): *A* MRI = 3 cm; LFB top image = 2 mm. LFB and PLP bottom images = 1 mm; inset control LFB = 300 μm; MS LFB and MS PLP = 150 μm. *D* = 200 μm; *G =* 50 μm; I LFB = 2 mm; CD20 = 20 μm; K NAWM/mDAWM = 50 μm; inset = 10 μm. B/W, back and white; DWM, deep white matter; PV, periventricular; SC, subcortical. Bar graphs are expressed as mean ± SEM, Box plot are expressed as mean and SD; *p < 0.05; ^##^ *p* < 0.0001.

Paraffin‐embedded brain blocks encompassing subcortical (Sc), deep white matter (dWM), and periventricular (PV) WM from 27 progressive MS and 5 control donors (Table 1) were provided by the Netherlands Brain Bank. No difference in the mean age at death (MS: 64.48 ± 2.54 years, controls: 64.00 ± 2.34 years; Welch's t‐test; t_(15.63)_ = 0.139; *p* = 0.891) and postmortem delay (MS: 9:25 ± 0:16 h/min; controls: 8:06 ± 0:54 h/min; Welch's t‐test; t_(4.665)_ = 1.420; *p* = 0.219) was present. The average MS case disease duration was 27.32 ± 2.65 years. The tissue inclusion was guided by the histological presence of areas of microscopic diffuse abnormality (Fig. 1). The whole study was performed in strict compliance with ethical requirements of the Amsterdam University medical centers/VU medical centrum, Amsterdam. Informed consent was asked to the donors.

**Table 1:** Summary of the cases used for the whole study.

Number	M/F	Age (years)	MS type	MS duration (years)	PMD (h:min)	Cause of death
MS1	M	86	SPMS	44	11:10	Pneumonia
MS2	M	56	PPMS	14	9:50	Exhaustion
MS3	M	80	SPMS	34	9:40	Pneumonia
MS4	M	58	SPMS	19	9:15	Pneumonia
MS5	M	71	SPMS	15	8:45	Carcinoma
MS6	M	60	SPMS	31	7:30	Pneumonia
MS7	F	53	SPMS	16	7:15	Euthanasia
MS8	F	87	SPMS	18	9:30	Renal insufficiency
MS9	F	48	SPMS	22	11:50	Respiratory failure
MS10	M	59	PMS n/a	n/a	10:45	n/a
MS11	M	66	PPMS	15	10:55	Euthanasia
MS12	F	76	SPMS	23	7:30	Pyelonephritis
MS13	M	57	SPMS	25	10:15	Sepsis
MS14	F	56	SPMS	23	10:30	Suicide
MS15	F	51	PPMS	15	9:45	Euthanasia
MS16	F	35	SPMS	10	10:20	Euthanasia
MS17	F	75	SPMS	46	9:45	Hematoma pons
MS18	M	70	SPMS	47	6:55	Heart failure
MS19	F	74	SPMS	47	7:50	Euthanasia
MS20	F	60	RRMS/SPMS	22	9:25	Euthanasia
MS21	M	54	SPMS	21	7:55	Euthanasia
MS22	M	82	SPMS	44	8:05	Pneumonia
MS23	M	50	SPMS	21	10:50	Euthanasia
MS24	F	77	SPMS	26	10:05	CVA
MS25	F	82	SPMS	60	8:35	Euthanasia
MS26	F	57	SPMS	25	10:40	Euthanasia
MS27	F	61	SPMS	n/a	10:00	Euthanasia
C1	M	62	CTRL	n/a	10:15	Adenocarcinoma
C2	M	70	CTRL	n/a	7:30	Pancreas carcinoma
C3	F	61	CTRL	n/a	10:15	Pneumonia
C4	F	69	CTRL	n/a	6:15	Cardiogenic shock
C5	F	58	CTRL	n/a	6:15	Multiple organic failure

C, non‐demented controls; CTRL, control; h:min, hours:minutes; MS, multiple sclerosis cases; n/a, not given; PMS, progressive MS; PPMS, primary progressive MS; RRMS, relapsing–remitting MS; SPMS, secondary progressive MS.

### Material preparation

Per case all available brain blocks were sliced in 10‐um‐thick slices, set on glass slides and deparaffinized (3 × 10 min in xylene, followed by ethanol 95%–70%, 5 × 5 min). Antibody details for immunohistochemistry are shown in Table 2.

**Table 2:** List of antibodies, dilutions, and suppliers.

Ab anti‐	Species	Dilution	Supplier	RRID/catalog number
PLP	Mouse	1:500	BioRad antibodies (US)	AB_2237198
LN3 (MHC‐II)	Mouse	1:500	Pierce (US)	AB_10979984
CD20 (L26)	Mouse	1:100	Dako (US)	AB_2282030
CD3 (F7.2.38)	Mouse	1:100	Dako (US)	AB_2631163
BCAS‐1 (NaBC1)	Mouse	1:500	Santa Cruz Biotechnologies (US)	AB_10839529
SMI312	Mouse	1:500	LabCorp Drug dev. (US)	AB_2566782
Lyso‐PC	Rabbit	1:100	Antibodies online (Germany)	ABIN7442600
MBP	Rabbit	1:200	Abcam (UK)	AB_881251
citMBP	Mouse	1:100	Merck (Germany)	AB_2920596
dMBP	Rabbit	1:200	US Biologicals (US)	M9758‐04

### WM assessment

Staining protocols using luxol fast blue (LFB),^5^, ^20^ antibodies against proteolipid protein (PLP) and major histocompatibility complex type‐II (MHC‐II) were used to assess the tissue presence of NAWM, mDAWM, WM lesions and remyelinating/remyelinated shadow lesions. For LFB staining, sections were incubated (58°C, overnight) in 0.1% LFB solution (Gurr, Electron Microscopy Sciences, Hatfield, PA, USA), further washed‐out in 96% ethanol and milli‐Q water (2–3 sec, and 3 sec, respectively), differentiated (0.05% lithium carbonate solution, 5 sec, Merck Millipore, Germany and 70% ethanol, 5–7 sec), dehydrated (Milli‐Q water followed by 96%–100% ethanol and xylene; 3–5 min, 2 × 5 min, 3 × 5 min, respectively), and coverslipped using Entellan (Sigma Aldrich, US). For immunohistochemistry experiments, anti‐PLP and anti‐MHC‐II primary antibodies were incubated after deparaffination, antigen retrieval (30 min, 95°C, Tris‐EDTA, pH 9.0, BDH, Germany), hydrogen peroxidase blockade (1–3% H_2_O_2_, Sigma Aldrich, US), and blocking solution steps (normal goat or donkey serum, Agilent, US; Jackson IR, US or bovine serum albumin, Sigma Aldrich, US; 3% in triphosphate buffer‐TBS 1x and 0.1% Triton‐X). After overnight incubation biotinylated secondary antibody application (1:400 in blocking solution 2 h) was followed by steps with avidin‐biotin complex kits (ABC; 15 min; Vector laboratories, US), diaminobenzidine (15 min; DAB +0.0015% H_2_O_2_, Agilent, US), hematoxylin staining (1 min; Fluka GmbH, Germany), and coverslip with Entellan. The same procedure applies to the other DAB‐stained markers, like CD3, lyso‐phosphatidylcholine, breast carcinoma amplified sequence‐1 (BCAS1, a marker for remyelinating oligodendrocytes^21^), and neurofilament (SMI‐312), with some adaptations.

### Fluorophore immunohistochemistry

To stain the tissue using fluorophores, during the second day of staining either a direct incubation with secondary fluorescent antibodies (Alexa 488 or 594, 1:400, ThermoFisher, US) or an incubation with biotinylated secondary antibodies followed by ABC/Envision kit (Agilent, US) and tyramide precipitation or streptavidin+tyramide protocols (in presence of 0.0015% H_2_O_2_, Tyramide Alexa 488 or 594, ThermoFisher, US) was carried out. All the slices underwent DAPI nuclear staining (1:1000, 5 min; Agilent, US) and were covered using mowiol (Sigma Aldrich, US) plus DABCO. To assess signal specificity during fluorophore and DAB experiments negative and positive (e.g., tonsil staining for CD20^+^ and CD3^+^ cell analysis) controls were added when appropriate.

### Image acquisition

Whole slides were first scanned (Vectra Polaris, Perkin Elmer inc., US) and digitalized for WM assessment and MHC‐II^+^ cell quantification. Same procedure was used for tissue stained with antibodies against CD20, MBP, citrullinated MBP, and degraded MBP (400× magnification). To quantify axonal density and the proportion citrullinated MBP/degraded MBP:MBP, images were taken using a DM5000 fluorescent microscope (NA 1.2; Leica Biosystems, Germany). For blister prevalence and the citrullinated MBP amount in MHC‐II+ cells, pictures were acquired using an SP8 inverted confocal microscope (NA 1.3; Leica Biosystems, Germany).

### Image analysis

To perform our analysis we selected the regions of interest (ROIs) from slide‐scanned images using Qu‐Path software^22^ and post‐process the whole images for quantification using Fiji Image‐J (NIH, US).

#### mDAWM quantification

mDAWM selection was performed screening LFB‐ and PLP‐stained slices (from MS and controls) under the microscope and digitally, searching for areas of ill‐defined borders of lower myelination in comparison with the surrounding NAWM. We excluded regions in the close proximity to lesions (<1 mm), guiding our selection using structural postmortem magnetic resonance imaging (MRI) performed before block excision.^23^ MRI inspection did not reveal the presence of classically defined‐DAWM in the region of excision or in the surroundings.^15^ Moreover, to reduce the artifact risk we considered as mDAWM only those areas whose surface was higher than 0.1 mm^2^ and excluding regions whose myelin density change could be otherwise defined by variation in fiber orientation. Myelin density quantification was performed adapting a previously used protocol,^20^ which involved ROI selection and manual segmentation to retain the maximum amount of pixel associated to myelin signal. To confirm the quality of the mDAWM selection, random digitally acquired ROIs of the tissue were automatically processed using the Image‐J algorithm *percentile* (Fig. 1) and the majority of the sections was further cross‐sectional inspected to estimate the mDAWM extension in tissue‐depth (data not shown).

#### MHC‐II, CD20, CD3, and BCAS‐1^+^ cell quantification

To quantify MHC‐II^+^ cells, we used the same ROIs selected for myelin quantification and assigned a score of 0 (no visible MHC‐II^+^ cells), 1 (1–5 MHC‐II^+^ cells), 2 (6–20 MHC‐II^+^ cells), or 3 (>20 cells MHC‐II^+^ cells). Density of CD20^+^, CD3^+^, BCAS‐1^+^ cells, and cluster of MHCII^+^ cells (nodules, identified following^24^, ^25^) was obtained by counting the number of positive cells/nodules, divided by the ROI surface (expressed as cells/μm^2^). Only cells presenting evident cellular membrane staining were counted.

#### Vein analysis in mDAWM

Vein^+^ mDAWM areas were visually classified looking for either an orthogonal or parallel inner vessel from which the mDAWM expands in at least two directions. Further inspections of the form of the vessel (e.g., tunica media thickness) were executed to exclude arteries/arterioles from analysis.

#### Wallerian degeneration analysis

Estimation of Wallerian pathology in mDAWM tissue was carried out acquiring a maximum of four random images (*xy* = 200*150 μm) from NAWM and mDAWM and analyzing them using Fiji Image‐J algorithms, surface analysis plug‐ins (percentage of area stained by antibodies against SMI312), and count plug‐ins (amount of axonal blebs‐blebs/μm^2^).

#### Myelin blister quantification

To quantify the blister‐, bleb‐ and axonal degenerative swelling‐prevalence we used a previously published protocol^5^ (with some adaptations), by which four random confocal images (*xyz*: 153.9*153.9*0.1 μm) were acquired and myelinated axons stratified according to predetermined criteria.^5^

#### LPC, cit/dMBP quantification

Quantification of lyso‐phosphatidylcholine, citrullinated/degraded MBP was obtained either acquiring a maximum of four images per case/region (*xy*: 200*150 μm, for lyso‐phosphatidylcholine) or equidimensional slide‐scanned images (*xy*: 1.25*0.96 mm, for citMBP and dMBP). Single axon analysis on the tissue stained with antibodies against MBP/citrullinated/degraded MBP was performed segmenting the images (*xy*: 200*150 μm) using a constant auto‐threshold algorithm, drawing ROIs around the margin of the analyzed myelinated axons and superimposing the channel associated to citrullinated/degraded MBP staining. Selected myelinated axons were later inspected for swelling presence and stratified, accordingly.

#### citMBP content in MHC‐II^+^ cells

To analyze the content of citrullinated MBP in MHC‐II^+^ cells a maximum of 4 confocal images (*xyz*: 387.88*387.88*0.1 μm) per case/area were acquired. Thereafter a random/blind selection of visible MHC‐II^+^ cells per image was followed by background subtraction and automatic segmentation. A skeletonize protocol and a skeleton script were run to stratify the sample according to its shape using previously reported criteria.^26^ Upon stratification of our sample in *ramified* (long processes, small cell body); *hyper‐ramified* (short processes, wide cell body); *rod‐like* (bipolar processes, large cell body), and *amoeboid* (macrophage‐like shape) cells we analyzed the presence of citrullinated MBP in the different categories (+ vs. −) and evaluated the degree of positivity analyzing the percentage of citrullinated MBP in the different cells. The latter was performed associating a level of citrullinated MBP positivity (+/− to +++) in MHC‐II^+^ cells according to the whole NAWM microglia sample percentiles (<25th, 25th–50th, 50th–75th, and >75th).

### Image presentation

For representative purposes some DAB stained images underwent color deconvolution using standard image‐J plug‐ins and representative fluorescent/confocal images have been cropped and brightness/contrast adjusted without altering the region comparability.

### Exclusion criteria

Slides whose staining was unsatisfactory were either restained or excluded from the study. Two cases from the original selection were excluded from final analysis due to the lack of satisfying clinical/histopathological assessment.

### Statistics

Parametric/nonparametric t‐tests (preceded by Shapiro–Wilk or Kolmogorov–Smirnov test), one‐way ANOVA or chi‐squared/Fisher's tests were used, when appropriate. In case of not‐homogeneous variance a ratio t‐test was used. ANOVA's were preceded by Spearman's and Bartlett's test for homoscedasticity and followed by multiple comparison post hoc correction. Correlation analysis was performed using Spearman's test. Data were analyzed using Prism 9.0 (GraphPad software, US) and considered significant when the *p*‐value was lower than 0.05.

## Results

### MS mDAWM shows limited expansion and signs of weak inflammation

In search of candidate regions to study early degeneration stages of MS WM we concentrated on microscopic regions of subtle myelin density reduction in the NAWM, and which we therefore dubbed as mDAWM. To assess the mDAWM we investigated LBF‐ and PLP‐stained brain tissue in a cohort of progressive MS cases (primary progressive *n* = 3; secondary progressive *n* = 15; average duration of disease 26.22 ± 3.33 years, Fig. 1A). In total we used 30 blocks that came from frontal (60%), temporal (30%), and parietal (10%) regions of 18 cases. Further exploration of material acquired from five controls showed no frank signs of mDAWM in non‐MS tissue.

The mDAWM typically presents with ill‐defined borders, variable LFB‐staining reduction, and small surface area (Fig. 1A,B; mean extension ROIs = 2.65 ± 0.29 mm^2^; range ROIs = 0.1–13.89 mm^2^; cutoff 0.1 mm^2^). Overall, myelin density analysis in the mDAWM reports a significantly reduced myelin staining (LFB) when compared with the matched NAWM and control WM (Fig. 1C). Furthermore, the analyzed mDAWM contains weak increase in MHC‐II reactivity (Fig. 1D,E) which negatively correlates with myelin density in the same region and no increase in MHC‐II^+^ clusters. These results are indicative of an augmented microglia/macrophage presence, as the count of CD20^+^ B‐lymphocytes did not differ between NAWM and mDAWM (Fig. 1F–J). Furthermore, no increase in the density of CD3^+^ T‐lymphocytes was found in the mDAWM when compared with NAWM (Fig. 1K,L).

### mDAWM in MS lacks remyelination, WD, and preferentially extends around inner veins

**Figure 2:**
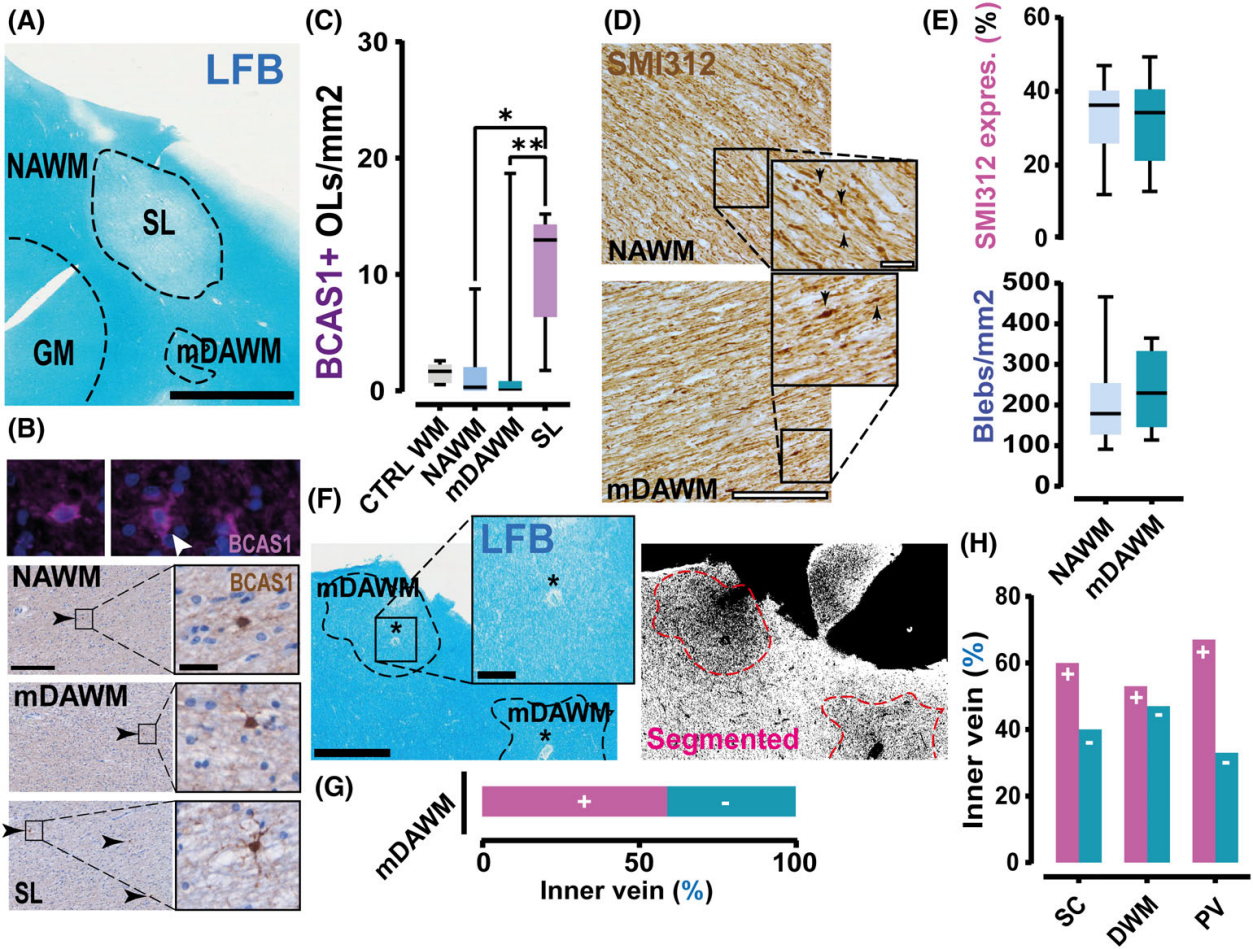
Lack of remyelination, Wallerian degenerative pathology, and expansion around veins of the mDAWM. (A) LFB image: presence of NAWM, mDAWM and a shadow lesion (SL) in an MS case. (B) Top panel: typical morphology of BCAS‐1^+^ remyelinating OLs in MS tissue. Bottom panel: expression of BCAS‐1^+^ OLs in NAWM, mDAWM and SLs. (C) Box plot shows the density of BCAS‐1^+^ cells in control (CTRL) and MS tissue (n control = 5; NAWM = 18; mDAWM = 18; SLs = 5; Kruskal–Wallis test; KW = 14.31; *p* = 0.002; Dunn's multiple comparison test; *p* = 0.286; WM control vs. mDAWM; *p* = 0.003; mDAWM vs. SLs; *p* = 0.99; NAWM vs. mDAWM). (D) Example images acquired from NAWM and mDAWM stained with SMI312. Insets: typical axonal swellings (blebs, arrows) both in the NAWM and mDAWM MS tissue. (E) Top box plot: axonal density analysis carried out in both NAWM and mDAWM regions of MS tissue (*n* = 7; 31.310 ± 4.333 vs. 32.640 ± 4.599%; Paired *t*‐test; *t*_(6)_ = 0.176; *p* = 0.866 NAWM vs. mDAWM). Bottom graph shows the count of blebs in the same material (*n* = 7; 209 ± 47.680 vs. 234.3 ± 35.33 blebs/mm^2^; Paired *t*‐test; *t*_(6)_ = 0.691; *p* = 0.515; NAWM vs. mDAWM). (F) Example of two allegedly distinct mDAWM regions that expand around a vein, seemingly located in the center of the area in an MS case. Left panel: LFB acquisition, right panel: automatic segmentation of the LFB image. Asterisk indicates the detected vein in the tissue. (G) Contingency graph shows a descriptive quantification of the inner vein + and − mDAWM in MS. (H) Bar graph shows the analysis of the inner vein +/− mDAWM after stratification based on location (n SC = 30; DWM = 40; PV = 6; Chi^2^ test for trend; Chi^2^_(1)_ = 0.013; *p* = 0.908; SC vs. DWM vs. PV). Scale bars: *A* = 4 mm; *B* fluorescent = 20 μm, DAB = 200 μm, inset DAB = 30 μm; D = 250 μm; inset = 50 μm; *F* = 1 mm; inset = 250 μm. OL, oligodendrocyte; Box plots report mean and SD; *p < 0.05; **p < 0.01.

To clarify whether the selected mDAWM was not the result of ongoing remyelination or Wallerian degeneration we studied the lesion morphology and the presence of newly myelinating oligodendrocytes (using the marker BCAS‐1). First, histologic appearance of the mDAWM showed substantial shape and boundary differences compared to MS shadow lesions (Fig. 2A). Second, the BCAS‐1^+^ cell density was similar between mDAWM and control tissues (NAWM and control WM), and significantly lower than remyelinating shadow lesions (Fig. 2B,C). Moreover, as shown in Figure 2D,E neither axonal density nor the estimation of axonal swellings (blebs) differed between mDAWM and NAWM, suggesting that the mDAWM is less likely the result of Wallerian pathology. Finally, the mDAWM showed a clear association (59% of total mDAWM analyzed, no difference between Sc‐, dWM, and PV WM) with the presence of inner veins (Fig. 2F–H), which share similarities with the well‐reported central vein present in MS WM lesions,^17^, ^18^ and which suggest the possible link between mDAWM and temporally evolving areas of frank demyelination.

### Increased prevalence of myelin blisters in the mDAWM

**Figure 3:**
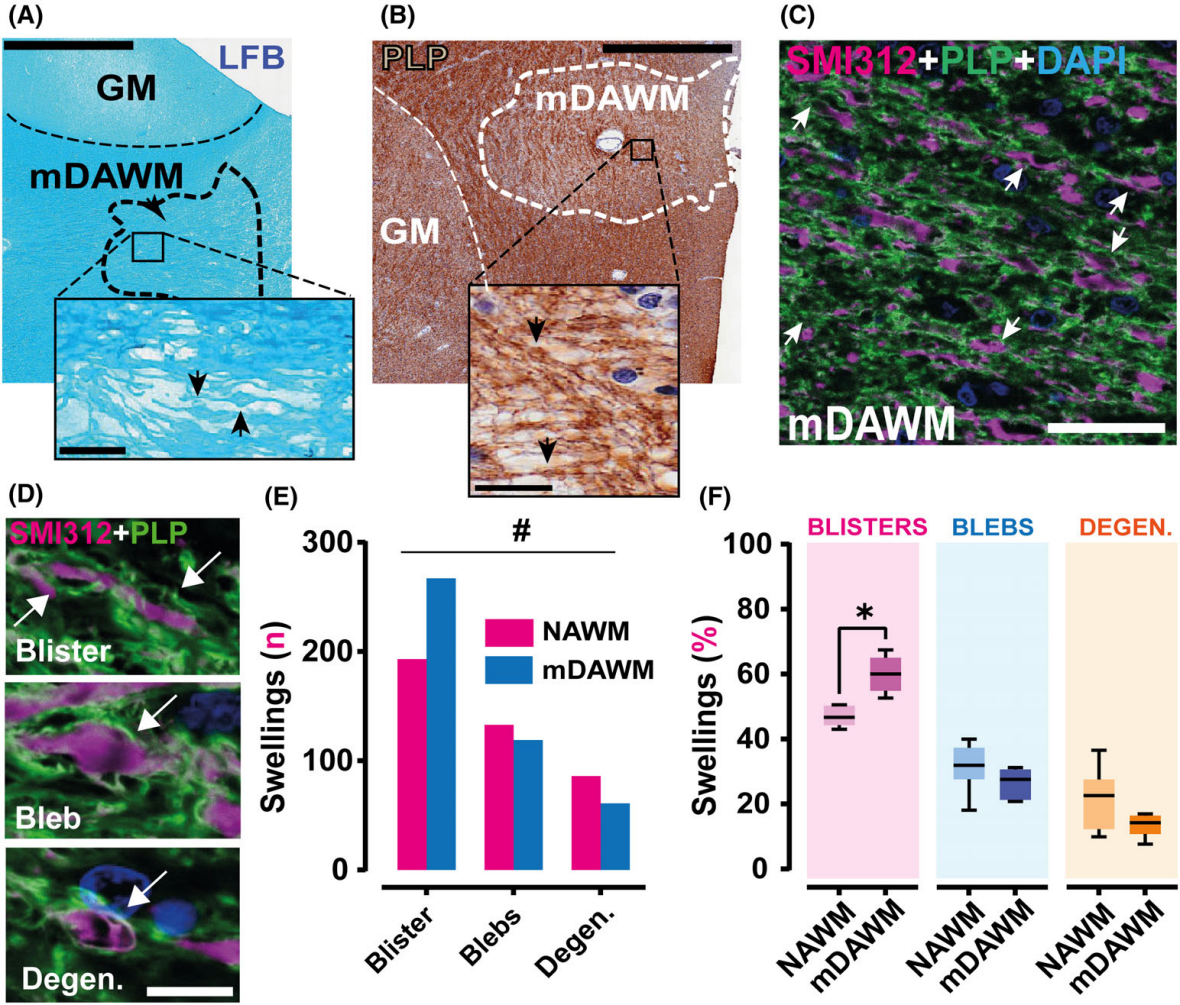
Myelin blisters in the mDAWM. (A and B) LFB and PLP images depict the presence of mDAWM swellings (arrow) in two cases used for analysis. (C) Double staining of mDAWM material with antibodies anti‐PLP and anti‐SMI312 shows the presence of several swelling processes indicative of axon‐myelinic imbalance (arrows). (D) Analysis of MS material retrieved the presence of different categories of myelin swellings. Top: *myelin blister* (myelin that detaches the axonal process); middle: axonal swelling (*bleb*); bottom: *axonal degenerative* processes inside swellings. Arrows indicate the different abnormalities. (E) Frequency distribution plot of the different classes of swellings shows a statistical difference between NAWM and mDAWM (n swellings NAWM = 412; n swellings mDAWM = 447; Chi^2^ test; Chi^2^_(2)_ = 15.53; *p* = 0.0004; NAWM vs. mDAWM) without difference in the density of analyzed total swellings (data not shown; 2409.0 ± 615.7 vs. 2548.0 ± 267.4 swelling/mm^2^ after correction for density myelination; paired *t*‐test; *t*_(5)_ = 0.185; *p* = 0.861; NAWM vs. mDAWM). (F) Box plot shows a specific increase in blister prevalence in mDAWM compared with NAWM (*n* = 6; 46.970 ± 1.261 vs. 59.990 ± 2.264%; Paired *t*‐test; *t*_(5)_ = 4.024; *p* = 0.010; NAWM vs. mDAWM). Scale bars: *A* and *B* = 800 μm, insets = 20 μm; *C* = 40 μm; *D* = 10 μm. Box plots report mean and SD; *p < 0.05; ^#^ *p* < 0.001.

To investigate whether the subtle myelin loss in mDAWM is associated with an exacerbation of the morphological alterations previously observed in the MS NAWM,^5^ we quantified the prevalence of myelin blisters in this area. Figure 3A,B depicts the presence of typical myelin swellings in the mDAWM. In line with our NAWM observations^5^ the mDAWM also hosts recognizable blisters, blebs, and axonal degenerative swellings (Fig. 3C,D). In total 859 myelin swellings were annotated and analyzed (NAWM: 412; mDAWM: 447). In accordance with our expectations, the mDAWM is characterized by 20% higher prevalence of blisters than in matched NAWM. This increase was not reported when quantifying axonal blebs and axonal degenerative processes (Fig. 3E,F) supporting myelin blister relevance in very early stages of reduced WM myelination.

### Phospholipid and MBP posttranslational modifications in the mDAWM

**Figure 4:**
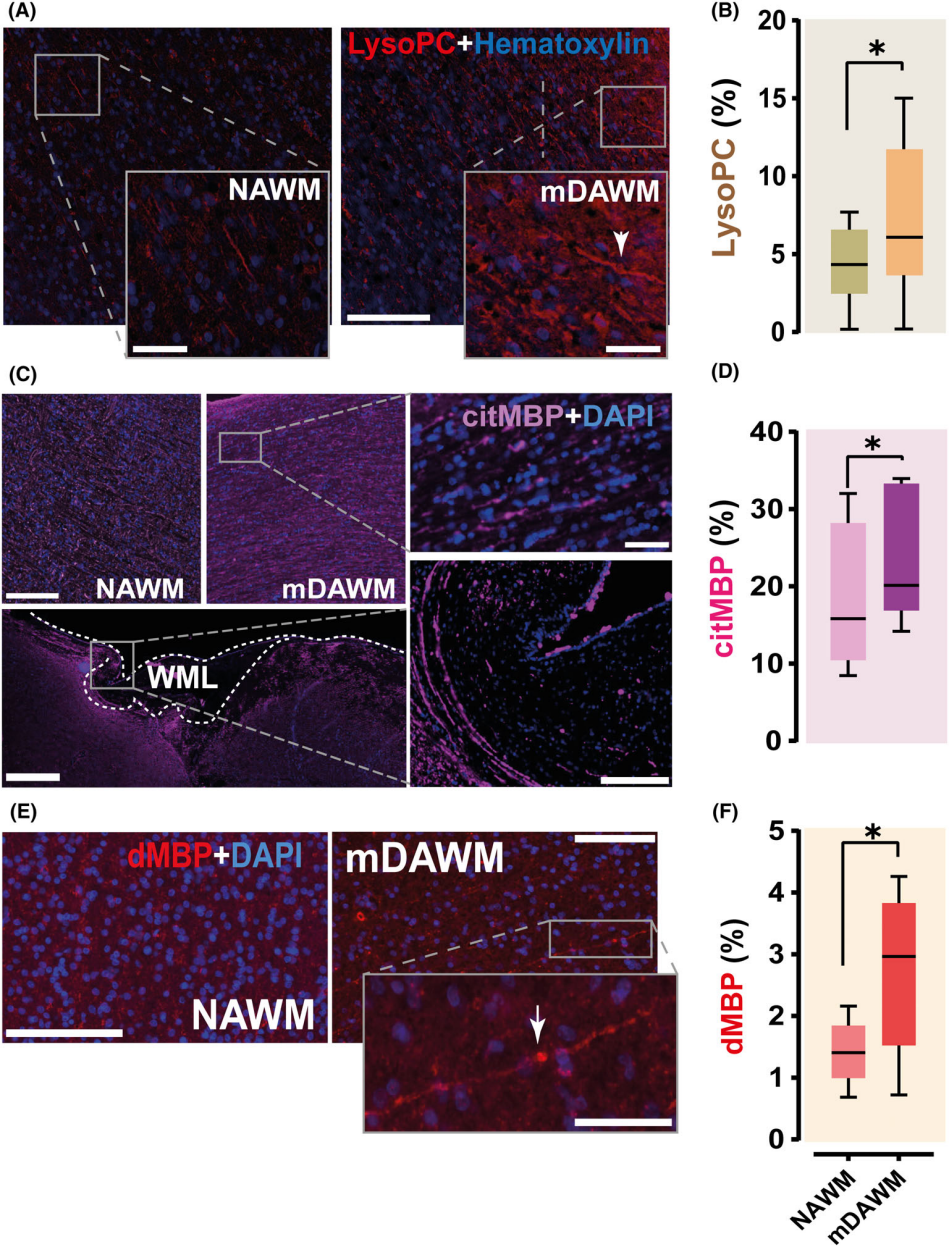
Phospholipid alterations and protein posttranslational modification in MS mDAWM. (A) Microscopic images showing lyso‐phosphatidylcholine (lyso‐PC) expression in MS NAWM and mDAWM. Arrow in the mDAWM inset points at a myelin swelling positive for lyso‐PC. (B) Box plot reports a significant increase of lyso‐PC reactivity in the mDAWM when compared with the NAWM of MS brains (*n* = 6; 4.324 ± 1.054 vs. 7.130 ± 2.103%; ratio paired *t*‐test; *t*_(5)_ = 2.634; *p* = 0.046; NAWM vs. mDAWM). (C) Examples showing the expression of citMBP in the NAWM and the mDAWM (top) and around a white matter lesion (WML, bottom). Insets shows a magnification of the regions. (D) Box plot shows an increased presence of citMBP in the mDAWM versus the NAWM in MS tissue, after correction based on the level of myelination of the regions (*n* = 5; 18.620 ± 4.255 vs. 24.090 ± 3.902%; ratio paired *t*‐test; *t*_(4)_ = 3.565; *p* = 0.023; NAWM vs. mDAWM). (E) Images showing the presence of dMBP in the NAWM and the mDAWM. Arrow in the inset points at a likely myelinated axon stained positive for dMBP. (F) Plot indicates a significant increase of dMBP in the mDAWM when compared with the NAWM of MS brains (*n* = 5; 1.415 ± 0.236 vs. 2.732 ± 0.594%; ratio paired *t*‐test; *t*_(4)_ = 4.243; *p* = 0.013; NAWM vs. mDAWM). Scale bars: *A* = 200 μm, insets = 50 μm; *C* NAWM and mDAWM = 200 μm, WML = 800 μm, inset above = 50 μm, below = 200 μm; *E* = 200 μm, inset = 100 μm. citMBP, citrullinated MBP; dMBP, degraded MBP; lyso‐PC, lyso‐phosphatidylcholine. Box plots report mean and SD; *p < 0.05.

Potential triggers for blister forming in MS NAWM are (i) prodromal phospholipid polarity alterations and (ii) augmented posttranslational modification at the level of MBP (e.g., citrullination).^5^, ^7^ Due to the increased blister prevalence in the mDAWM we expected that these mechanisms are also more apparent in this region when compared with the NAWM. To clarify these aspects we first stained our material with antibodies against lyso‐phosphatidylcholine, a highly polar membrane phospholipid whose conversion from phosphatidylcholine is instigated by a Ca^2+^‐mediated translocation of cytoplasmic phospholipase 2. Lyso‐phosphatidylcholine was found to induce demyelination when externally infused in mouse brains^27^ and it can alter membrane curvature, making it a candidate for myelin blister formation.^28^ Analysis of the overall expression of lyso‐phosphatidylcholine revealed its augmented presence in MS mDAWM versus the NAWM (Fig. 4A,B) where it is also associated with clear myelin swellings (arrow in inset Fig. 4A). Besides, a documented effect of lyso‐phosphatidylcholine conversion is also the higher activation of the protein arginine deiminase enzyme PAD2, which is responsible of myelin protein citrullination.^10^ Accordingly, analysis of our samples following citrullinated MBP staining showed an increase in the presence of this posttranslationally modified form of MBP in the mDAWM versus the NAWM (Fig. 4C,D). This effect is accompanied by a similar, albeit more modest, increase in degraded forms of MBP (reminiscent of myelin debris) in the mDAWM when compared with the NAWM (Fig. 4E,F). These findings hint at an exacerbation of MS‐relevant chemical changes at the level of myelin proteins prior to myelin fragmentation in the mDAWM.

### Citrullinated MBP in the mDAWM associated with swellings and active microglia response

**Figure 5:**
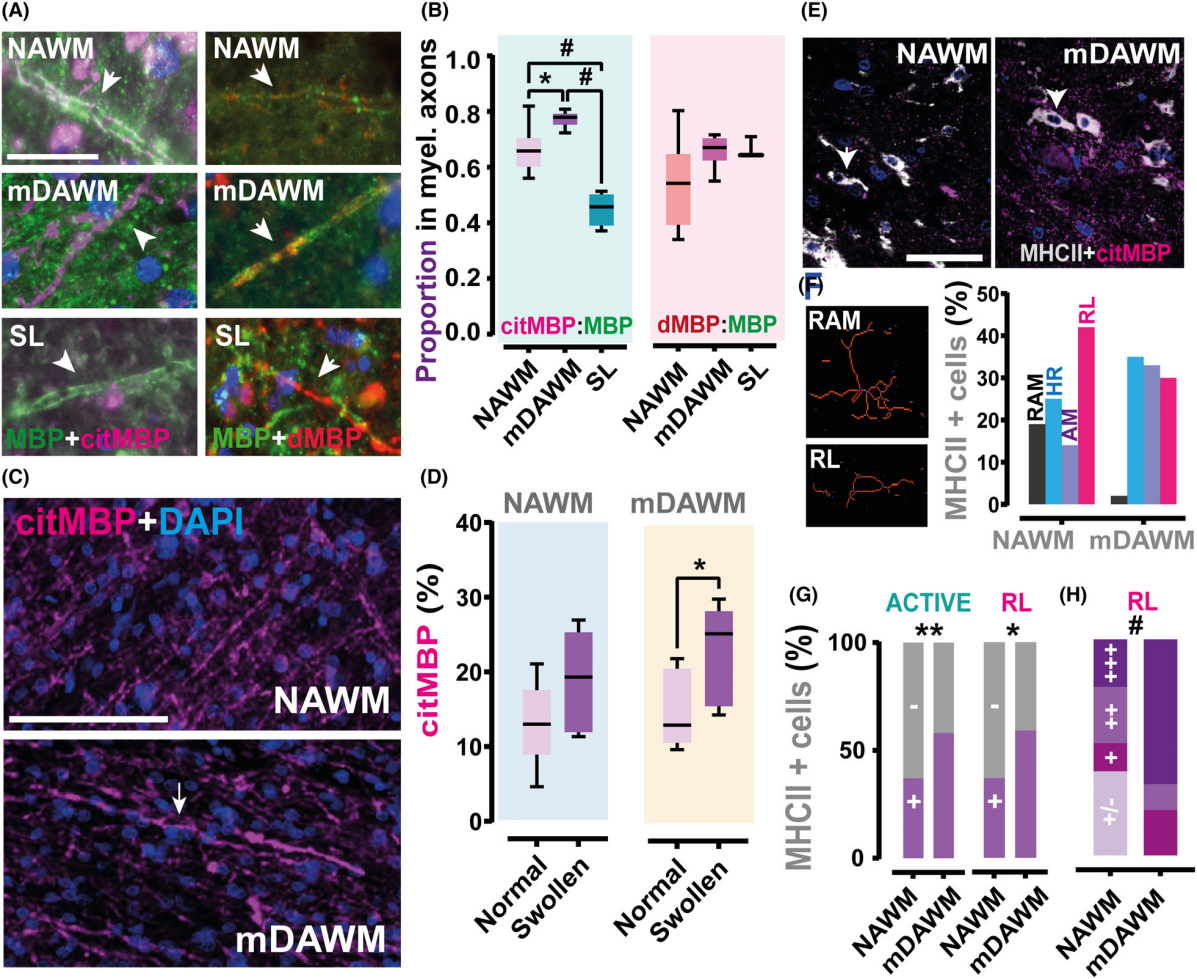
Higher content of citMBP in myelinated axons and MHC‐II^+^ cells in the mDAWM. (A) Fluorescent microscope acquisition of single myelinated axons (indicated by arrows) stained with antibodies against normal and citrullinated/degraded MBP in NAWM, mDAWM and SLs from an MS case. (B) Plots showing the proportion citMBP:MBP and dMBP:MBP in the mDAWM when compared with MS NAWM and MS SLs (CitMBP: n NAWM = 9; mDAWM = 6; SL = 4; proportion = 0.665 ± 0.026 vs. 0.773 ± 0.012 vs. 0.450 ± 0.030; One‐way ANOVA; *F*_(2,16)_ = 31.89; *p* < 0.0001; Tukey's multiple comparison test; *p* = 0.013 NAWM vs. mDAWM; *p* < 0.0001 NAWM vs. SLs; *p* < 0.001 mDAWM vs. SLs; dMBP: n NAWM = 9; mDAWM = 6; SL = 3; proportion = 0.534 ± 0.050 vs. 0.655 ± 0.024 vs. 0.660 ± 0.022; Kruskal–Wallis test; KW = 4.337; *p* = 0.113; NAWM vs. mDAWM vs. SLs). (C) Representative images of myelinated axons staining positive for citMBP in the NAWM and the mDAWM. Arrow indicates a myelin swelling. (D) Analysis stratifying the sample in seemingly normal myelinated axons (normal) and axons containing a swelling (swollen) shows that in the mDAWM swollen axons are characterized by higher citMBP content than normal processes (NAWM: *n* = 5; 13.070 ± 2.308 vs. 18.960 ± 2.621; ratio paired *t*‐test; *t*_(5)_ = 2.288; *p* = 0.071; normal vs. swollen processes; mDAWM: *n* = 5; 15.240 ± 2.337 vs. 22.720 ± 2.989; ratio paired *t*‐test; *t*_(4)_ = 4.546; *p* = 0.010; normal vs. swollen processes). (E) example of MHCII^+^ cells that present with citMBP content in both the NAWM and the mDAWM of an MS case. Arrowheads indicate active microglia cells. (F) Left image shows the typical appearance of a ramified (resting, top image), and a rod‐like microglia (active, bottom) after skeleton analysis. Graph reports the percent of different microglia cells analyzed in both NAWM and mDAWM. (G) proportion of citMBP positive and negative active (overall) and rod‐like MHCII^+^ cells in the NAWM and the mDAWM (Active: Fisher's exact test; *p* = 0.0011; NAWM vs. mDAWM; rod‐like: Fisher's exact test; *p* = 0.0435; NAWM vs. mDAWM). (H) Graph showing that more RL cells in the mDAWM are extremely positive (+++) for citMBP than in the NAWM (Chi^2^ test for trend; Chi^2^_(1)_ = 11.40; *p* = 0.0007; NAWM vs. mDAWM). Scale bars: *A* = 100 μm; *C* = 100 μm; *E* = 50 μm. AM, amoeboid microglia; citMBP, citrullinated MBP; dMBP, degraded MBP; HIP, hyper‐ramified microglia; RAM, ramified microglia; RL, rod‐like microglia. Box plots report mean and SD; *p < 0.05; **p < 0.01; ^#^ *p* < 0.001.

Aiming at a sharper look at myelin protein citrullination in prodromal phases of myelin pathology in MS mDAWM we further performed an analysis on single myelinated axons (Fig. 5A) in the mDAWM. As shown in Figure 5B the proportion of citrullinated/normal MBP is significantly altered in the mDAWM, suggesting that a greater surface of myelinated axons hosts such modified form of MBP in comparison to axons in the NAWM. Intriguingly, the same analysis at the level of comparable axons in the shadow lesions showed an inversion of the citrullinated MBP presence in this area, endorsing a putative specific role of MBP citrullination in the early degenerative/prelesional stages in MS. Similar analysis did not show a clear increase in degraded MBP at axonal level, arguing that the previously observed increase in degraded MBP in the mDAWM might be indeed attributed to debris of degenerated myelin constituents (Fig. 5B).

Interestingly, while previous studies from our group did not highlight specific associations between enhanced NAWM citrullination and blister/swelling formation in MS^5^ (confirmed here), this effect was clearly prevalent in the mDAWM (Fig. 5C,D), hinting at an intimate relationship between the decompaction of myelin sheaths, presumably caused by the sustained citrullination of MBP, and the increased blistering in the mDAWM.

Finally, to test whether the enhanced presence of citrullinated MBP in the mDAWM can form a possible trigger of reactive microglia states against myelin constituents we investigated the topical association between MHC‐II^+^ microglia (although we cannot exclude that this marker labels monocyte‐derived macrophages) with this altered form of MBP in NAWM and mDAWM. A sample of 286 MHC‐II^+^ cells (NAWM: 148; mDAWM: 138) was randomly selected, and further stratified using skeleton scripts in ramified (slightly active) and active (Fig. 5F). As expected, our selected samples contained more ramified cells in the NAWM (18.9%; 28 out of 148) than the mDAWM (2.2%; 3 out of 138). The overall amount of active microglia was further classified into hyper‐ramified (NAWM: 25%; 37 out of 148; mDAWM: 34.8%; 48out of 138), amoeboid (NAWM: 14.2%; 21 out of 148; mDAWM: 33.3%; 46 out of 138), and rod‐like cells (NAWM: 41.9%; 62 out of 148; mDAWM: 29.7%; 41 out of 138, Fig. 5F). Overall, the mDAWM contains more citrullinated MBP^+^ cells than the NAWM (NAWM: 40%; mDAWM: 57%). Analysis stratifying the sample to include only active MHC‐II^+^ cells confirmed this increase in the mDAWM (NAWM: 37%; mDAWM: 58%, Fig. 5G) where a significant difference was particularly spotted when comparing the hyper‐ramified (NAWM: 41%; mDAWM: 65%) and rod‐like cells (NAWM: 37%; mDAWM: 59%, Fig. 5G). Interestingly, when stratifying our sample based on the level of positivity to citrullinated MBP (from weakly positive to extremely positive), only rod‐like MHC^+^ cells in the mDAWM had a different distribution than in the NAWM (Fig. 5H), being associated with higher content of this modified form of myelin proteins. Overall, this result support an active microglia engagement in the process of highly immunogenic myelin debris phagocytosis in the mDAWM.

## Discussion

The work in this publication builds on the *inside‐out* concept, which states that MS starts in the CNS and that autoimmunity is the consequence of the (excessive) release of (posttranslationally modified) myelin fragments.^1^, ^2^ Earlier work revealed presence of myelin blisters in MS brain areas devoid of immune‐related aberrations.^5^ In this study we go further back on the pathology timescale to gain insight into factors contributing to myelin blistering.

Although studies employing imaging,^29^ microscopy,^5^, ^30^, ^31^, ^32^, ^33^ and biochemistry techniques,^5^, ^7^, ^34^ have highlighted the abnormal nature of the NAWM in MS brains, the relevance of such aberrations for the formation of overt lesions remains unknown.^1^ Here we describe the presence of axo‐myelinic unit‐relevant destabilization features outside the NAWM limits, where small regions of diffuse abnormality (mDAWM), characterized by modest tissue inflammation^35^, host an exacerbation of such events. Compared with the NAWM, the mDAWM shows (i) myelin blister increase, (ii) enhanced expression of polar phospholipids, (iii) increased presence of citrullinated/degraded MBP, and (iv) heightened phagocytosis of posttranslationally modified myelin constituents. These findings, together with the absence of Wallerian degeneration, a hallmark of the classically defined DAWM,^15^, ^16^ and the association with a vein located in the innermost part of the area, a feature similar to that of the central vein in WM lesion formation,^17^, ^18^ make of the mDAWM a likely separate (very) early pathological aspect of MS brains able to recapitulate the pathophysiological cascade of events leading toward more frank demyelination stages. This latter concept aligns with recent investigations into the DAWM of progressive MS patients^36^, ^37^ and, more importantly, with the abovementioned *inside‐out* hypothesis of MS origin.^1^, ^2^, ^5^

Although high‐throughput live‐cell imaging studies are needed to confirm this sequence of events, we interpret the correlation between high MHC‐II^+^ scores and myelin reduction in the mDAWM as supportive of the inside‐out paradigm of MS origin. In fact, in our specimens only the highest score (Score 3) seems to clearly associate with more severe myelination drops, an effect accompanied by the lack of significant difference between the amount of MHC‐II^+^ cells in the NAWM versus the mDAWM. This finding, combined with the lack of a specific increase of both B and T lymphocytes in the mDAWM when compared with NAWM, contradicts the hypothesis that a massive primary recruitment of inflammatory cells causes tissue demyelination, proposing that heightened microglia reactivity secondarily intervenes to clear up myelin debris when demyelination processes have already started. Supportive of this notion is our result on citrullinated MBP microglia/macrophage phagocytic activity (see below), which might precede the formation of the well‐reported nodules of microglia.^24^ In line with this possibility we did not retrieve a substantial increase of such nodules in mDAWM when compared with NAWM. Interestingly, this feature is in line with studies on other (neuro)degenerative diseases, like atherosclerosis and Alzheimer's disease, which are characterized by primary tissue damage and secondary inflammatory response operated by immune cells.^38^, ^39^

Due to the low emergence of new lesions in advanced secondary progressive MS cases,^4^, ^40^ the late disease stage/age of our cohort may represent an argument against the association between mDAWM and WM lesion formation. Although more investigations are warranted, our conclusions are in line with extensive histopathological examinations by Luchetti *et al*. which observed sustained inflammatory and demyelinating activity several decades after the diagnosis in late stage progressive MS cases,^41^ and by studies on living secondary progressive subjects that highlighted a clear evolution overtime from DAWM to focal WM lesions^37^ which correlates with the clinical progression of the disease.^36^

Our myelin blister results further support the prelesional/early lesional nature of mDAWM. Myelin blisters are emerging as important MS pathology correlates, being spotted in several NAWM and peri‐lesion areas of MS brains^5^ and, more recently, in the optic nerve,^33^ a region often associated with the earliest MS lesion formation.^42^ Nonetheless, in line with the early involvement of myelin blisters in lesion formation, studies employing cuprizone mouse models of MS have reported the presence of similar morphological alterations in mice weeks prior to demyelination.^30^

Although the precise mechanistic dissection of blister formation in MS brains and their relevance for the mDAWM origin are still under investigation, we speculate that early destabilizing events at the level of axon‐myelinic unit may involve altered nodes of Ranvier elongation and Na^+^ abnormalities,^5^ substantiated by augmented Na^+^ concentrations in both NAWM^43^ and DAWM^44^ of MS patients. These aberrations may trigger an uncontrolled myelinic Ca^2+^ entrance^6^ which exacerbates the pathological biochemical myelin sheath changes. Notably, augmented Ca^2+^ might activate the formation of lyso‐phosphatidylcholine, a highly polar phospholipid,^45^ which, in turn, reduces the amount of Ca^2+^ needed to stimulate PAD‐2 activity,^9^ which catalyzes myelin protein citrullination.^46^ Interestingly, studies reported the ability of lyso‐phosphatidylcholine infusions in mice to promote myelin blistering^27^ and the presence of prodromal lipid polarity abnormalities in MS NAWM.^7^ In accordance with these findings, we observed an enhanced lyso‐phosphatidylcholine and citrullinated MBP reactivity in mDAWM tissue when compared with the NAWM of MS brains, a concept that corroborates the early myelin biochemical change exacerbation in the evolution toward overt states of lesion. Intriguingly, myelin protein citrullination, largely present in MS brains and meninges^5^, ^47^ is shown to precede demyelination and autoimmune reaction in cuprizone mouse model experiments,^11^ drive the progression of CNS pathology in experimental autoimmune encephalitis in marmoset monkeys,^48^ trigger the detachment of myelin lamellae,^49^ and contribute to render the myelin debris more immunogenic.^12^

**Figure 6:**
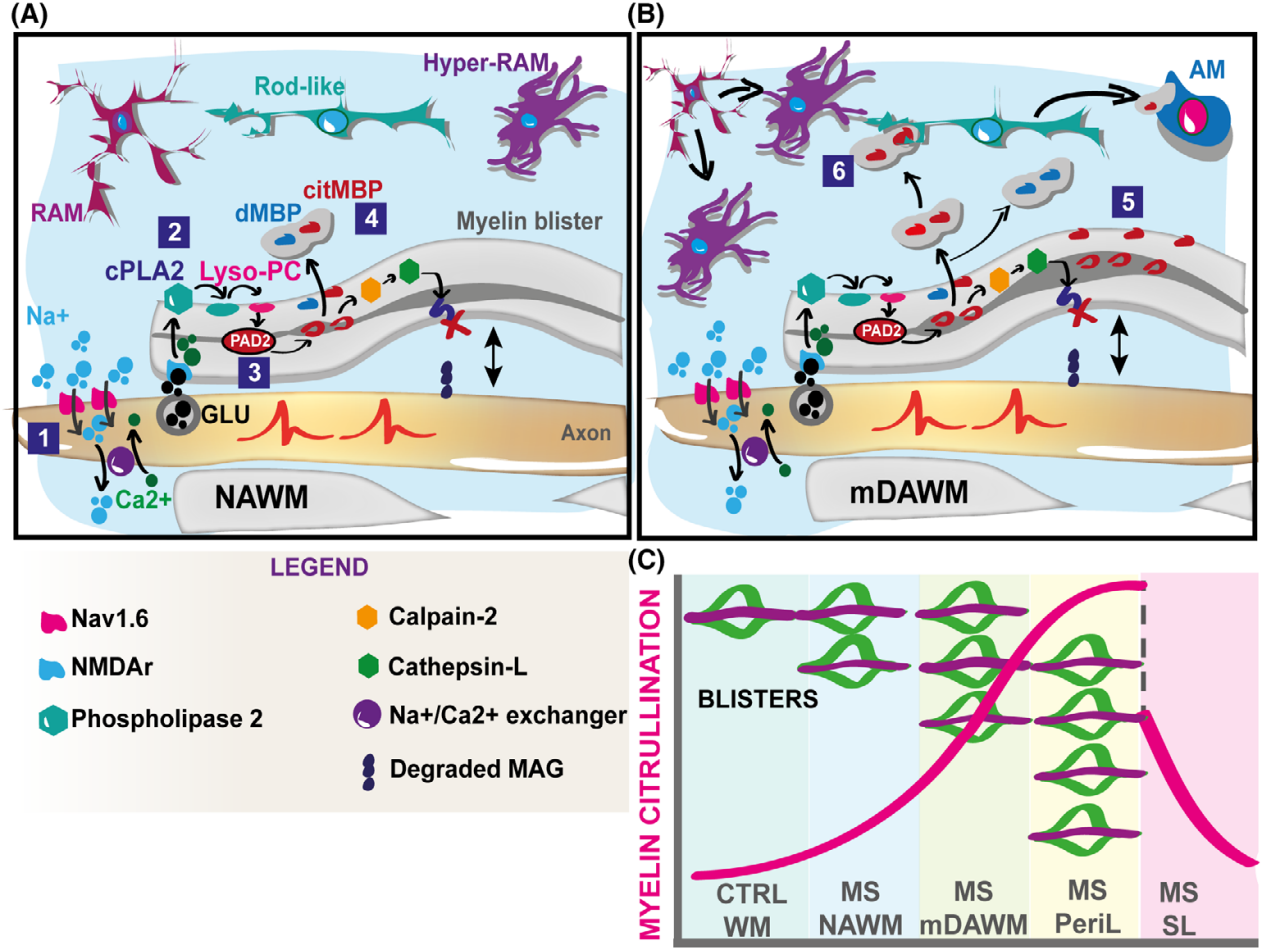
Mechanistic model. (A) Schematic depiction of the cascade of pathological events involved in myelin blister formation in the NAWM, as proposed in Luchicchi *et al*.^5^ with some adaptations. (1) Altered Na^+^ homeostasis produces an aberrant Na^+^ influx in dysfunctional nodes of Ranvier in MS. Consequently, the Na^+^/Ca^2+^ exchanger might be pushed to work in a reverse fashion^8^ leading to increase of axonal Ca^2+^ and higher release of glutamate to the myelin. (2) Over‐stimulation of NMDArs produces Ca^2+^ overload in the myelin, instigating the translocation of cPLA_2_ with conversion of lyso‐PC from PC. (3) Lyso‐PC reduces the Ca^2+^ necessary to activated the PAD2 with consequent citrullination of MBP and activation of the calpain‐cathepsin axis (4). Cathepsin‐L degrades the MAG promoting the formation of myelin blisters. (B) In the mDAWM an exacerbation of the cascade of mechanisms instigates progression of deterioration with consequent heightened prevalence of blistering of the AMS and higher citrullination of the MBP and myelin debris (dMBP). More citMBP and dMBP are released as myelin constituent debris (5) against whom rod‐like microglia (6) is particularly involved in engulfing the PTM proteins. (C) Schematic overview of the sequence of degenerative processes moving from the WM of healthy controls (as previously reported using F95 α‐peptidylcitrulline antibodies^5^) to MS NAWM, mDAWM, peri‐lesional WM and remyelinating SLs. Citrullination of myelin proteins follows a gradual increase until lesions are formed to reduce its presence in case of remyelination. At the same time myelin blister prevalence increases when MS WM lesions are forming. citMBP, citrullinated MBP; cPLA2, cytoplasmic phospholipase A2; dMBP, degraded MBP; Glu, glutamate; HIP, hyper‐ramified microglia; MAG, myelin associated glycoprotein; Nav1.6, voltage‐gated sodium channel 1.6; NMDAr, n‐methyl‐d‐aspartate receptor; PAD‐2, protein arginine deiminase‐2; RAM, ramified microglia.

Although the enlargement of lamella spacing may promote states of intra‐myelinic oedema,^50^ representing a confounding factor for our study, we tend to disregard this circumstance in our mDAWM dataset. In fact, our observations do not align with the tissue sponginess previously reported in oedemas,^50^ and the expansion and form of the retrieved blisters/swellings share remarkable similarities with those previously found in the NAWM.^5^ On the other hand, the citrullinated MBP‐dependent sheath detachment potentially makes the myelin more vulnerable to protease attack,^51^ an aspect involved in degraded myelin‐associated glycoprotein formation^14^ and, allegedly, blister formation.^5^ Therefore, it is tempting to speculate that via an increased myelin citrullination in the mDAWM, substantiated by the here reported stronger association between citrullinated MBP and swelling formation in the same tissue than in the NAWM, more blistering and more myelin degeneration is deciding the fate toward WM lesion in MS brains. In response to this a secondary immune reaction might intervene, strongly triggered by citrullinated myelin constituents (Fig. 6A,B). In line with this, heightened microglia/macrophage activity in the mDAWM presents with phagocytic activity against citrullinated MBP. Interestingly, after morphology‐based stratification, we found a particular reactivity of rod‐like‐shaped microglia cells in the mDAWM against citrullinated myelin. Although the function of these specific cell types is still misterious,^52^ recent studies have underlined their role in the transition from ramified forms of microglia to an amoeboid macrophage‐like activation state,^53^ together with their phagocytic features,^54^ and their scarce involvement in pro‐inflammatory cytokine production.^55^ Therefore, a parsimonious interpretation of our results might involve a prompt conversion of inactive state microglia into actively phagocytic phenotypes in response to the increase in highly citrullinated production of myelin debris in the mDAWM. This interpretation aligns with studies showing rapid in vitro transition of this microglia type into amoeboid states when exposed to lipopolysaccharide,^55^ although it cannot exclude the property of rod‐like‐shaped microglia to shift back to a ramified/less active state.^56^, ^57^

To conclude, in this study we highlight an exacerbation of the pathological hallmarks which we previously spotted in the NAWM of MS brains,^5^ showing that, via an (i) increase of posttranslational myelin modifications (which are likely specific for prelesional stages of MS WM, Fig. 6C), (ii) myelin blister enhancement and (iii) microglia‐directed response toward citrullinated MBP, the NAWM might encounter a degenerative evolution toward microscopic diffusivity, here dubbed as mDAWM. This evidence represents a valuable aspect to dissect the cascade of prelesional events that leads to the formation of focal lesions in MS brains, and to characterized early degenerative WM areas to intervene early in progressive forms of MS, for which limited pharmacological approaches are available.

## Author Contributions

AL, GMG, ES, JP, and GJS: Study design and data analysis. GMG, TSH, FU, CF, LvD, PMB, and MS: Performed experiments, data acquisition, and data analysis. BtH and JJG: Study design and critical revision. AL and GJS: Wrote the manuscript with input from all the authors.

## Funding Information

Funding for this study were obtained by Stichting MS Research, MoveS, Klimmen tegen MS (MS16‐945b, awarded to JJG), donor campaign Stichting Leef! (awarded to AL), Ammodo KNAW (awarded to JJG), National MS society (Progressive MS alliance award, PA2021‐36033 awarded to JJG and AL), and Dutch National MS Foundation (Research grant OZ2021‐008 awarded to AL). PMB obtained funding from Stichting MS Research (MS19‐1049). TSH obtained funding from Nederlandse Organisatie voor Wetenschappelijk Onderzoek (NWO 2022/SGW/01291477).

## Conflict of Interest

Authors declare no potential conflict of interest directly related to this manuscript. E.S. obtained unrelated speaker's fee from Novartis and Merck.

## Data Availability

Raw data can be provided upon reasonable request to other investigators for the purpose of replication, only.
